# Experience of Autistic Children and Their Families During the Pandemic: From Distress to Coping Strategies

**DOI:** 10.1007/s10803-021-05233-z

**Published:** 2021-08-27

**Authors:** Claudine Jacques, Geneviève Saulnier, Agnès Éthier, Isabelle Soulières

**Affiliations:** 1 Present Address: Department of Psychoeducation and Psychology, Université du Québec en Outaouais, 283 Alexandre Taché Blvd, Poste 2272, Postbox 1250, Gatineau, QC J8X 3X7 Canada; 2 Centre intégré de sante et des services sociaux de l’Outaouais, Gatineau, QC Canada; 3 Department of Psychology, Université du Québec à Montréal, Montreal, QC Canada; 4 CIUSSS du Nord-de-l’Île-de-Montréal, Hôpital en sante mentale Rivière-des-Prairies, local SU-1335, 100, rue Sherbrooke Ouest, Montréal, QC H2X 3P2 Canada; 5 Present Address: Hôpital Pierre-Janet - Pavillon Juvénile, 20, rue Pharand, bureau 1106, Gatineau, QC J9A 1K7 Canada

**Keywords:** Autism, Children, Family, COVID-19, Special interests, Social isolation

## Abstract

To understand the perspectives and needs of autistic children and their families in the context of an emergency, 109 parents and 56 autistic children (5.75–18 years) from Canada completed an online survey about needs, barriers and facilitators to coping with the pandemic. Parents’ concerns about their child’s development and difficulties managing their child’s behaviors before and during pandemic were significantly associated. Parents identified maintaining social relationships and implementing appropriate interventions to their child’s characteristics as facilitators during the pandemic. Both children and parents identified lack of socialization as a main difficulty. Among children, 92.9% associated electronic devices with their well-being. This study highlighted the need to consider the child’s autistic characteristics and interests to implement emergency accommodations and services.

**Supplementary Information:**

The online version contains supplementary material available at 10.1007/s10803-021-05233-z.

The World Health Organization (WHO) declared COVID-19 a pandemic in March 2020, bringing challenges around the world. For about 3 months, the entire province of Quebec, like all other Canadian provinces, was locked down, resulting in the complete closure of schools and childcare services, as well as drastically reducing the possibilities of support, both from professionals and relatives. Adaptations were necessary to deal with this situation for the entire population (Bueno-Notivol et al., 2021; Chanchlani et al., 2021; Fegert et al., 2020), but particularly for children with special needs and their families (Aishworiya & Kang, 2020; Dhiman et al., 2020; Nonweiler et al., 2020).

From the start of the pandemic, both researchers and clinicians feared that autistic children and their families would face many challenges, such as dealing on their own with pandemic-related stress (Eshraghi et al., 2020; Pellicano et al., 2020; Spain et al., 2021). These families had to reorganize their daily lives, they encountered barriers in accessing healthcare and specialized services, and the children experienced many educational changes (Aishworiya & Kang, 2020; Ameis et al., 2020). Given the variability of autistic characteristics across children, as well as across developmental stages (Kendall et al., 2013; Wallace et al., 2019), many questions remain about how to organize services to meet the needs and particularities of autistic children and achieve a better adaptation while complying with public health guidelines.

As suggested by the family adaptation theory, when a family is exposed to a stressful situation, it tries to cope with reality. Therefore, family is part of a system that has an impact on it, and special attention must be paid to all the components of the system, namely all the contexts in times of crisis (Luthar et al., 2000; McCubbin et al., 1999; Pauzé & Petitpas, 2013; Shaffer et al., 2002). Given some characteristics of autistic children (e.g., difficulties anticipating changes or presence of anxiety) (APA, 2013; Simonoff et al., 2008), special consideration must be given to them and their families, in order to better adapt services to their specific needs.

To have a comprehensive understanding of the needs of autistic children and their families, we had to first identify both the factors that make adaptation easier (facilitating factors) and those that hinder adaptation (barriers) during a pandemic. Therefore, the goal of our study was to gain a better understanding of the needs of autistic children and their families in the context of a pandemic by identifying facilitating factors and barriers to make recommendations that could help guide services. We considered it important to learn about the perspectives not only of the parents, but also of the autistic children themselves. Therefore, we surveyed parents of autistic children as well as their children about their own needs and perceptions. More precisely, the objectives of our study were (1) to identify the factors that hinder or facilitate daily functioning during a pandemic; (2) to identify the coping strategies to be implemented to ensure the children’s quality of life, as well as the family’s adaptation; and (3) to recommend practices, support mechanisms, and services to be offered by the community, institutions, and governments at a time when further waves of the pandemic were predicted.

## Method

### Research Design

The use of a mixed research design, combining both qualitative and quantitative data, helped provide a comprehensive understanding of the needs and perceptions of autistic children and their parents facing the COVID-19 pandemic. To collect the data, we created a questionnaire to be completed through LimeSurvey. To obtain a variety of respondent profiles, we also offered the option to complete the questionnaire by interview. The survey took place between July and October 2020. The participants provided informed consent through the online platform or by email. This study was approved by the research ethics committee of Université du Québec en Outaouais.

### Participants

Participants were recruited through advertisements disseminated by professional and parents’ associations specializing in autism and on social networks. Two groups of participants completed the new French-language *Questionnaire on the Needs of Autistic Children and Adolescents During the Pandemic*. One hundred and nine parents of autistic children (88 boys, 19 girls, and two non-binary) aged between 2.6 and 18.10 years (15.6% aged 2 to 6, 50.5% aged 7 to 12, and 34% aged 13 to 18) completed the questionnaire (three parents by phone interview and 106 online) and 56 children (52.8% of all the children concerned by the study; 46 boys, nine girls, and one non-binary) aged 5.75 to 18 years filled out a subsection of the same questionnaire. All the participants lived in Quebec, a French-speaking province of Canada. The inclusion criteria were (1) autism diagnosis for the child, (2) sufficient knowledge of French to answer the questionnaire, (3) child aged 24 months to 18 years (see Table 1 for the sociodemographic characteristics).

**Table 1:** Sociodemographic characteristics

	*n*	%
Family income		
Less than 29,999$	15	13.76
30 000 to 49,999$	12	11.01
50 000 to 69,999$	10	9.17
70 000 to 89,999$	19	17.43
90 000 to 1,19,999$	18	16.51
1,20,000$ and more	29	26.61
I’d rather not answer	6	5.50
Level of education of parent 1		
Secondary school not completed	4	3.67
Secondary school	13	11.93
College (technical program)	28	25.69
Baccalaureate	37	33.94
Master	18	16.51
Doctorate	2	1.83
Post-doctorate	2	1.83
Other	5	4.59
Level of education of parent 2		
Secondary school not completed	10	9.17
Secondary school	22	20.18
College (technical program)	30	27.52
Baccalaureate	24	22.01
Master	7	6.42
Doctorate	2	1.83
Post-doctorate	1	0.92
Other	3	2.75
Not applicable	8	7.33
I’d rather not answer	1	0.92
Missing	1	0.92
Number of siblings		
None	18	16.51
One	45	41.28
Two	28	25.69
Three	8	7.34
Four	4	3.67
Missing	6	5.51
Diagnoses in siblings		
Yes	38	34.86
No	70	64.22
I’d rather not answer	1	0.92
Educational setting of the autistic child		
Regular class	49	44.95
Specialized class	26	23.85
Other	18	16.51
N/A	16	14.68
Respondent’s gender		
Woman	102	93.58
Man	7	6.42
Place of residence		
Rural area	25	22.94
Urban area	84	77.06
Place of birth of parent 1		
Canada	94	86.23
France	10	9.17
Other	5	5.59
Place of birth of parent 2		
Canada	89	81.65
France	3	2.75
Algeria	2	1.83
Other	5	4.59
Missing	10	9.17
Child’s place of birth		
Canada	75	68.80
France	4	3.67
Algeria	2	1.83
Other	3	0.92
Missing	25	22.94
Family structure		
Two-parent family	69	63.30
Single parent family	28	25.69
Stepfamily	11	10.09
I’d rather not answer	1	0.92

This table demonstrates the sociodemographic data of the participating families (n=109). Family income is the annual family income in Canadian dollars. The number of siblings is in addition to their autistic child

### Questionnaire

Since no questionnaire existed for evaluating the target concept, we created one. Following a gray literature (INESSS, 2020; MEDPENB, 2020; NCTSN, 2020; UH, 2020; Waters et al., 2020) and scientific literature review (Narzisi, 2020; Twoy et al., 2007; White et al., 2013) about autistic children and their families in an emergency, a list of facilitating factors (n = 24) and barriers (n = 11) was generated by the principal investigator and a research assistant. Next, a first version of the questionnaire, which included 10 sections with open and forced-choice questions, was proposed. This preliminary version of the questionnaire was revised in an iterative process by two authors (specialized in neuropsychology and social work), two psychoeducational clinicians, and two graduate neuropsychology students, including one of the authors (AE). The questionnaire was then piloted with three parents of autistic children (preschool age, school age, and adolescent). The sections of the questionnaire remained the same, but changes were made to the wording of the questions and some items were added.

The final version of the questionnaire comprised multiple-choice and open-ended questions about sociodemographic and clinical characteristics, prepandemic situation, pandemic situation, barriers (n = 17), facilitating factors (n = 30), access to information and services during the pandemic period, perception of the pandemic situation, accommodations, and adaptive strategies. The child section concerned stress and difficulties caused by the pandemic, strategies for facing the pandemic and making it less stressful, and activities to help cope with the pandemic. Parents were informed that their child could read the questions and write the answers on their own or have access to their help. The survey pertained to the pandemic, more specifically the period of confinement and physical distancing in Spring 2020.

### Analyses

The final raw data were imported from LimeSurvey and analyzed with SPSS 25 software. Descriptive statistics were used to provide a general picture of the participants’ experiences (stress experienced by parents, facilitating factors, barriers, and children’s perceptions). Three sets of Pearson correlations were performed. First, we wanted to verify the relationship between difficulties managing the child’s behaviors and having concerns about the child’s development during the pandemic and the level of pandemic-related stress experienced by the family. Second, we assessed the relationship between difficulties managing the child’s behaviors and having concerns about the child’s development before and during the pandemic. Third, we assessed the relationship between these two variables and the facilitating factors or the barriers. Finally, a last set of correlations were performed with the same target variables and the sociodemographic and clinical characteristics. Also, thematic content analyses of the open-ended questions on facilitating factors, barriers, parents’ recommendations and children’s perceptions were carried out by identifying themes and subthemes and associating them with examples (Bardin, 1993; Roberts et al., 2019). An intercoder reliability- IRC of 87% (O’Connor & Joffe, 2020) was obtained by two research assistants on 20% of the randomly selected verbatim (ranging in the 10–25% suggested) (O’Connor & Joffe, 2020). In the event of a discordant category assignment, consensus was reached through discussion with two of the authors (AE and GS). The ICR ensured the reliability of the coding and categorization of nominal data. The ICR also made it possible to generate knowledge from a different and complementary reading of the data and to ensure maximal validity of the thematic analyses, which was particularly important in our multidisciplinary research team (Mukamurera et al., 2006; O’Connor & Joffe, 2020).

## Results

The results were grouped into six sections: (1) stress experienced by parents during the pandemic, (2) factors related to difficulties with behavior management and parents’ concerns during the pandemic, (3) facilitating factors, (4) barriers, (5) children’s perceptions, and (6) parents’ recommendations. Throughout this section, the names of the participants have been replaced by codes consisting of the letter “P” for “parent” or “C” for “child” and their ID.

### Stress Experienced by Parents During the Pandemic

Almost half of our respondents (45.9%) considered themselves anxious by nature, while only 16.5% reported handling stressful situations quite easily. Also, 52.3% of the parents reported having several strategies to cope with difficult situations.

Most parents (93.5%) considered the pandemic a highly stressful period for them ranging from: sometimes (43.1%), often (43.1%), or always (7.3%). When asked about the whole family, 91.7% of the parents identified the presence of high pandemic-related stress in their family ranging from: sometimes (50.5%), often (33.9%), or always (7.3%). According to 88.9% of the parents, the pandemic became less stressful over time: sometimes (38.5%), often (44.0%), or always (6.4%). A proportion of 67.9% of the parents indicated that the pandemic situation became more stressful ranging from: sometimes (54.1%), often (10.1%), or always (3.7%). No moderate or strong correlations involving parent-reported stress were found. See Table 1 in Supplementary Material.

### Factors Related to Behavior Management and Parents’ Concerns During the Pandemic

There was a moderate correlation between the variables “Child's behaviors were easy to manage on a daily basis before the pandemic.” and “Child's behaviors are easy to manage on a daily basis during the pandemic” (r = 0.519, p < 0.05). There was also a moderate correlation between the variables “being concerned about the child’s development before the pandemic” and “being concerned about the child’s development during the pandemic” (r = 0.534, p < 0.05). See Table 2.

**Table 2:** Associations between having concerned about the child’s development before and during the pandemic and child's behaviors are easy to manage on a daily basis before and during the pandemic

	1	2	3	4
1	–			
2	.534^**^	–		
3	− .322^**^	− .318^**^	–	
4	− .338^**^	− .503^**^	.519^**^	–

This table demonstrates the associations between being concerned about the child’s development*,* ease of managing behaviors before and during the pandemic (n = 109)

*p < .05 **p < .01

1. I was concerned about my child's development

2. I am concerned about my child's development

3. My child's behaviors were easy to manage on a daily basis

4. My child's behaviors are easy to manage on a daily basis

No strong or moderate correlations were found between the variables “being concerned about the child’s development” or “Child's behaviors are easy to manage on a daily basis” and the characteristics of the child, of the parents, or of the family. See Table 2 in Supplementary Material.

**Fig. 1:**
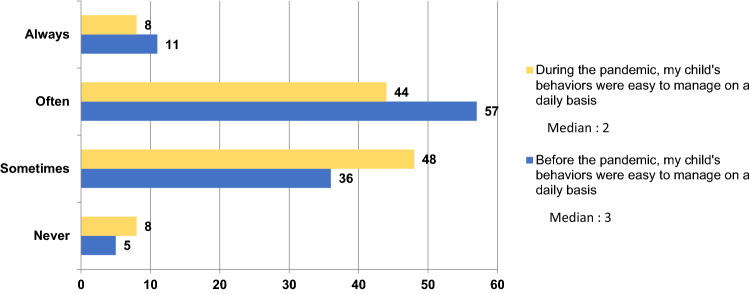
Differences in child’s behaviors before and during the pandemic. This figure shows the answers to the question: “My child’s behaviors are/were easy to manage on a daily basis” during the pandemic (in yellow) versus before the pandemic (in blue). Parents answered on a four-point Likert scale, from never (1; never find easy to manage their child’s behaviors) to always (4; always find easy to manage their child’s behaviors). The lower median during the pandemic suggests that the parents felt their child’s behaviors were less easy to manage on a daily basis during relative to before the pandemic

**Fig. 2:**
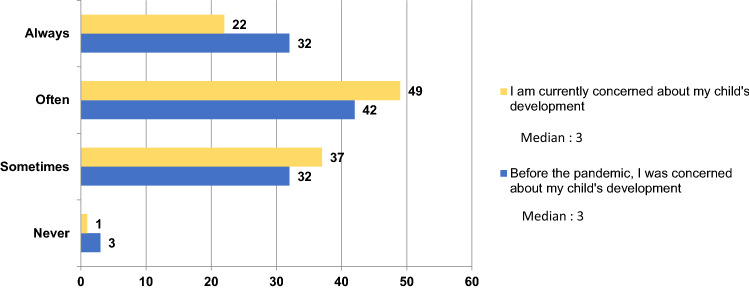
Differences in parents’ concerns about their child’s development before and during the pandemic. This figure shows the answers to the question: “I am/was concerned about my child’s development” during the pandemic (in yellow) versus before the pandemic (in blue). Parents answered on a four-point Likert scale, from never (1; never concerned about their child’s development) to always (4; always concerned about their child’s development). The medians show similar central tendencies in the answers before and during the pandemic, parents being “often” concerned about their child’s development

No strong or moderate correlations were found between the variables “being concerned about the child’s development” or “child's behaviors are easy to manage on a daily basis” and the facilitators and the barriers (see Tables 3 and 4 in Supplementary Material). See Figs. 1 and 2 for the differences in difficulties managing the child’s behaviors and parents’ concerns about their child’s development before and during the pandemic.

### What were the Facilitating Factors?

Data from the three subgroups (preschool, school age, and adolescent) were analyzed together because of the similarities in facilitating factors among the groups (see Supplementary Table 5 for top 3 facilitating factors by age group). Out of a list of 30 individual, family, and environmental facilitating factors, the parents reported which were the most helpful in facing the pandemic. The four most endorsed factors were: *understanding of their child’s needs* (73.4%), ranked first for individual factors among the three subgroups; *establishment of a routine with their child* (67%), ranked 1st in the school age and adolescent subgroups and 2nd in the preschool group for the environmental factors; *good communication between parents* (62.4%), ranked 1st in the preschool and adolescent subgroups and 2nd in the school age subgroup; and *possibility of spending time together* (61.5%), ranked 1st in the school age and 2nd in the preschool and adolescent subgroups. See Table 3 for the most important facilitating factors and Supplementary Table 6 for all the facilitating factors.

**Table 3:** Top five facilitating factors

Facilitators	*n*	%
I understand my child's needs	80	73.39
Establishing a routine with my child	73	67.00
Good communication between parents	68	62.39
The possibility of spending time together	67	61.47
My child is able to take care of himself	57	52.29

This table demonstrates the facilitating factors during the pandemic, as endorsed by the respondents (n = 109) in multiple-choice questions. The respondents could select all the answers that they thought had a positive impact on their capacity to cope with the pandemic

In the open question about facilitating factors, the parents made 55 statements. Three major themes emerged through the thematic analysis: (1) use of adapted interventions (n = 38, 69.1%), (2) COVID-19 management (n = 7, 12.7%), and (3) having access to informal (conjugal, parent, extended family, and friend) and formal (specialized and organized assistance) support (n = 4, 7.3%).

Parents identified the need to use adapted interventions to respond to their child’s specific needs. In this regard, many parents highlighted the importance of setting a routine (“Making sure to have a daily experience anchored in a routine similar to before the pandemic.”^1^—P73). They also mentioned using materials suitable to autistic characteristics (“A heavy blanket… really helped to calm the anxiety. He always has it with him.”—P2) and respecting their child’s needs and limitations (“I spoke with him to know what he needed.”—P173).

The parents managed the COVID situation with their children by giving them information and answering their questions (“Talking with him, without hiding reality too much and reassuring him.”—P228). They also downplayed the situation (“Having an attitude of downplaying events. Without denying the impact of COVID, not talking about it constantly and thinking about it all the time.”—P73).

Informal support and formal support were helpful for the families. Parents highlighted the importance of having access to specialized services (e.g., rehabilitation center for autistic children and professionals, such as psychologists, psychoeducators, occupational therapists, speech therapists, pediatrician, psychiatrist, and family doctor) for their children but also for themselves (“Having the chance to consult my psychologist to vent and help me continue.”—P172).

### What were the Barriers?

Out of a list of 17 barriers, parents reported which were the most difficult to face during the pandemic and which hindered their child’s adaptation. The three main barriers were: their child having too much access to electronic devices (56%), which was respectively the 1st and the 2nd obstacle in the preschool age and adolescent subgroups; being isolated from their relatives (55.1%), which was respectively the 1st and the 2nd obstacle in the preschool and school age subgroups; and having to pursue academic goals (while schools were closed; 51.4%), which was respectively the 1st and the 3rd obstacle in the adolescent and school age subgroups. See Table 4 for the most important barrier; Supplementary Material Tables 7 and 8 for barriers by age group and all the barriers respectively.

**Table 4:** Top five barriers

Barriers	*n*	%
My child has access to electronic devices	61	55.96
We are more isolated from our loved ones	60	55.05
It is necessary to pursue academic goals at a distance	56	51.38
My child focusses on his particular interests	53	48.62
My child has too much free time	52	47.71

This table demonstrates the barriers during the pandemic, as endorsed by the respondents (n = 109) in multiple-choice questions. The respondents could select all the answers that they thought had a negative impact on their capacity to cope with the pandemic

In the open question on barriers to their child’s adaptation during the pandemic, the parents made 52 statements. When the statements of the three groups were thematically analyzed together, four major themes emerged: (1) closure of specialized resources (n = 11, 21.2%), (2) lack of opportunities for social interactions (n = 9, 17.3%), (3) inadequate school support (n = 6, 11.5%), and (4) challenging behaviors (n = 5, 9.6%).

The closure of specialized resources was perceived as very difficult, with sometimes dramatic consequences. Autistic children and their parents felt isolated, given the lack of access to specialized services (“We were left completely alone and without resources, services, or respite. My child completely decompensated and we couldn’t even help him, and nobody could help us either.”—P165). Some children went through a period of regression (“The children regressed in their already-limited social skills.”—P167).

Parents also identified a significant impact of the pandemic on their child’s social interactions. Hence, they reported a lack of social interaction opportunities for their child (“The thing that is hardest for my child is that he can’t have friends over for his birthday because they don’t understand doing social distancing.”—P72). Parents also found their child to be lonely (“My child was more withdrawn and a little less communicative.”—P156; “He feels alone, he doesn't see much of the family.”—P222; “He is often sad because of feeling lonely and friendless and I find it difficult to help him in this regard.” –P34).

The support services and teaching opportunities offered by schools varied greatly during the pandemic. Some parents reported that school support was not adapted to the particularities of autistic children (“Students integrated in regular classes who were not failing were not targeted for individualized support [vulnerable is not only when it shows in the academic statistics and scores].”—P46).

Many challenging behaviors were also reported, particularly those related to anxiety (“It was hell. Tantrums, generalized anxiety: fear of dying, of choking, of breaking a bone, etc.”—P58) and to opposition (“A lot of violence with his little brother.”—P58).

### Children’s Perceptions

Among the children concerned, 51.4% (n = 56) filled out the questionnaire by themselves or with the help of their parents (to read the questions and/or to write the answers). In this subgroup, 78.6% mentioned that the pandemic was stressful for them ranging from: sometimes (28.6%), often (28.6%), or always (21.4%), whereas 80.7%% of the parents identified the presence of elevated stress in their child ranging from: sometimes (43.1%), often (23.9%), or always (14.7%). In terms of facilitating factors, 92.9% of the children indicated that electronics were what made them feel good during the pandemic. In an open-ended question about what helped reduce their stress, more than a quarter of the responses were about electronics (“lots of Minecraft and YouTube”—C79, “listen to YouTube videos” —C94, “play videogames”—C156, “play with my computer at Hello Neighbor and Nintendo switch at Mario Maker”—C176). The two other most important factors, board games and reading, were identified by 37.5% and 35.7% of the children, respectively. When the children were asked in an open-ended question what they would recommend to a stressed friend during the pandemic, 29% of all the recommendations were related to the social sphere, the main theme of this qualitative analysis. In 70% of cases, these recommendations pertained to communication and to closeness with their parents (“To talk to his mother to get help”—C111, “Talking to his parents”—C222), whereas the rest concerned visiting family and talking to trusted adults (“If he has a trusted adult, tell him he is stressed”—C34, “Go see the family”– C143).

As highlighted by the parents, the lack of socialization (namely “not seeing my friends” –C185) was identified as an important barrier by 57.1% of the children, among a list of seven potential barriers. Next came the fear of catching the virus (48.2% of the children) and the change in routine (35.7% of the children). A quarter of all the answers to the open-ended question about what they found difficult during the pandemic also pertained to the limitation of social interactions (“not see my family and friends”—C185, “not being able to spend time with my grandparents”—C78, “having to go to high school and not being able to see my school, my friends, and my teachers [from elementary school] one last time”—C171).

### Parents’ Recommendations for Service Organizations and Governments to Increase Support to Autistic Children and Their Parents During a Pandemic

Parents were asked what they would recommend for public services in the context of a pandemic. Parents made statements about what could be improved in specialized services (n = 33) and respite (n = 14), in schools (n = 31), and by the government (n = 4). In this section, they again stressed the lack of socialization (n = 21) as an important factor to consider.

### Specialized Services

There was a consensus among the parents regarding the importance of having access to services for all autistic children (“At the very least, we would have liked the services that support the families who have a child with ASD to be considered essential services.”—P2). The importance of access to experts in autism was also stressed by many parents (“Access to psychoeducators”—P133, “Easy consultation with a physician or psychologist specialized in autism”—P61). Access to specialized services during the confinement varied among the children. Parents indicated that it should be a priority to maintain basic services in various forms, such as online interventions or phone support (“It would have been relevant to continue the support even remotely, because we no longer had any services, e.g., the educator was calling once a week, but we would have liked the special education technician to be more present.”—P2). However, considering the heterogeneous profiles of autistic children, these services were not always suitable for meeting their variable needs. Sometimes, direct interventions were necessary but unavailable (“I would have needed more in-person visits from the caseworkers, notably from the intervention center, rather than just talking on the phone for 2 to 3 months. I was really all alone.”—P58). Finally, the importance of having access to respite was stressed repeatedly by parents (“There has to be respite for the families and targeted interventions by qualified personnel to help the families. At the time, there was literally nothing and we were left to ourselves without support of any kind.”—P181).

### School

The main theme that emerged was the importance of establishing a routine and of better monitoring with help from the teacher and other professionals from the school, as well as the implementation of adapted academic work (“Supply tools to help set up a routine, and not just worklists.”—P94). The second theme was the importance of follow-up by the school professionals (“That the caseworkers who already know the child continue their calls.”—P94). Finally, access to specialized counselors and maintenance of in-person schooling for children in specialized classes was the third theme identified by the parents (“Back to school for ASD classes in a regular school.”—P39).

### Government

Parents highlighted the importance of having access to consistent information. Some autistic children obtained information directly from the media. Constant changes in the information provided about the pandemic and lockdown had a negative impact on them. The parents of autistic children reported the need for accurate information about the virus, but also about health measures implemented in society, work, schools, and preschools (“Avoiding making government announcements that change quickly like the non-resumption of classes in September and then the total opposite a few days later.”—P142).

Parents also underlined the need for financial compensation, especially to have access to respite during periods of confinement (“Even the government could have helped by offering money to at least get respite. I was just left on my own and I had parental anxiety and exhaustion.”—P93).

## Discussion

In this study, we documented the experience of autistic children and their families during the pandemic using a survey of parents, who shared their experience and made recommendations on how to improve services in response to the COVID-19 pandemic. We also administered a subsection of the questionnaire to their autistic children, to better understand their specific needs and perceptions. To our knowledge, there is only one other study where autistic children were directly surveyed about their perceptions of the pandemic (Garcia et al., 2020).

The results from the parents' section showed that most of the sample considered the pandemic to be a very stressful time and that, for half of the sample, this was the case "often" or "always." There was a moderate correlation between concerns about the child’s development or difficulties managing the child’s behaviors before and during the pandemic. There were no correlations between the facilitating factors/barriers and concerns about the child’s development or difficulties managing the child’s behaviors during the pandemic. However, three main facilitators were identified by the parents in multiple-choice and open-ended questions: understanding their autistic child’s characteristics, implementing appropriate interventions, such as setting up a routine, and maintaining social relationships. In addition, too much access to electronics was identified as one of the main barriers.

More than three quarters of the children also considered the pandemic to be a stressful time, with half of them finding it often or always stressful. Like their parents who pointed out that social isolation was a barrier, the children identified the lack of socialization as a main barrier and emphasized the importance of a routine. However, their view was opposite to that of their parents regarding access to electronics. Indeed, the children reported how access to electronics and to their interests (e.g., board games, reading) contributed to their well-being.

Finally, the survey also allowed parents to share their suggestions by responding to the following question: what practices or services could have been set up, in particular by childcare centers, schools, workplaces, healthcare centers, community organizations, and the government, to promote your autistic child’s adaptation during the pandemic? First, parents stressed the importance of maintaining access to specialized services for autistic children and that services for autistic children should be considered essential. These services could take various forms: direct interventions (e.g., access to respite), telehealth interventions, or phone support. Second, parents highlighted that schools must ensure regular and targeted follow-ups with autistic children and offer specialized services if necessary. Third, they underlined that the government should provide accurate information about COVID-19 and the measures available for autistic children and their parents. Finally, parents indicated that all environments must be flexible to the reality of families with autistic children and must take into account autistic characteristics.

### What we Learned from this Research: Support for Autistic Children and Their Parents, Social Interactions, and Accommodations for Autistic Interests

Three elements were consistently highlighted by the parents and the children: the importance of supporting autistic children and their parents, the need for social interactions, and the place for autistic interests. Beyond the current pandemic, understanding these factors is important for better guiding interventions for autistic children.

### Supporting Autistic Children and Their Parents

As in previous studies where many parents of autistic children reported an increase of stress and anxiety during the pandemic (Adams et al., 2021; Alhuzimi, 2021; Calvano et al., 2021; Corbett et al., 2021; Spinelli et al., 2020), almost all our sample of parents considered this period to be a highly stressful time to varying level. A general consensus emerged among the parents on the importance of having access to information to understand their autistic child’s characteristics and to put in place measures suited to these characteristics. The pandemic has shown that interventions for autistic children must not only target the children directly, but also provide parents with information and tools to better understand and support their child through “parent-mediated interventions” (Ameis et al., 2020, p. 6). In our study, setting up a routine was a main facilitating factor identified by the parents and a helpful factor reported by the children. Previous studies on the pandemic have also highlighted the importance of setting up a routine, organizing free time, and structuring activities (Ameis et al., 2020; Narzisi, 2020; Neece et al., 2020). The importance of establishing a routine was identified by parents and children in both multiple-choice and open-ended questions, as well as by parents in the recommendation section. As with typical children (Fegert et al., 2020), another facilitating factor consisted in giving accurate information to the children about the pandemic. Since autistic children can sometimes have difficulty understanding what is happening (Eshraghi et al., 2020; Narzisi, 2020), it is necessary to adapt the information to make it accessible to various autistic profiles (e.g., use of visual representations, Kunda, 2013; Kunda & Goel, 2008).

Also, in line with our findings about the importance of giving accurate information, Suffren et al. (2021) highlighted the importance of providing complete information in order to reduce the anxiety of typical children. Indeed, like in our study, in which the “fear of catching the virus” was the second barrier identified by autistic children, Suffren et al. (2021) also showed a high level of similar fear in typical children. Here again, to help autistic children understand the pandemic, it is necessary to use tools that are suited to them and, to do this, parents need to be supported and well informed.

The situation was more difficult for families with autistic children who were struggling before the pandemic. Our study showed that the pandemic amplified parents’ concerns and exacerbated difficult behaviors already present in the children. Similarly, Colizzi et al. (2020) further observed that autistic children with pre-pandemic behavior problems were twice as likely to have more frequent and more intense behavior problems than before. Using a French sample similar to ours, Berard et al. (2021) also showed an aggravation of behavior problems during the pandemic. These findings also highlight the importance of ongoing support for the interventions that parents can implement in their children’s daily lives, even outside of a national emergency (Aishworiya & Kang, 2020; Ameis et al., 2020). In this regard, services should be increased for autistic children who are already experiencing difficulties. Yet, parents reported losing services during the pandemic and having little access to support (e.g. specialized services, classes, respite, medical support; Ameis et al., 2020; Eshraghi et al., 2020; Jeste et al., 2020). In short, it is imperative to ensure the continuity of autism interventions, propose direct and indirect intervention methods, and adapt the modalities and intensity of interventions to the child’s profile.

### The Need for Social Interactions

During the pandemic, social isolation had many negative consequences for adults and children in the general population. Isolation and loneliness were the main barrier identified by typical teenagers in a study by Tardif-Grenier et al. (2021) with a sample from the same area as the one in our study. Oomen et al. (2021) reported more drastic changes for non-autistic persons than for autistic persons regarding their “social life” (p. 13). Furthermore, it is not surprising that the caregivers and families of autistic children in our study felt isolated during the pandemic, as this was reported by others as well (more than 70% of the sample in Salt et al., (2020). Parents also stressed the importance of socialization in their autistic children’s lives. Our results highlighted a paradoxical aspect of autism: whereas it is commonly believed that this condition is indissociable from significant social deficits, the lack of social interactions was the main obstacle reported by autistic children as well as by their parents during the pandemic. For example, a parent shared that: “In our case, the source of distress is the lack of a network of true friends. For my child, this need is even stronger than for me, he is sad.”—P34. This finding was consistent with “loss of social contact” characterized by the “stress of not being able to see the loved ones” identified by autistic adults in Oomen et al. (2021, p. 11).

Although there is an abundant literature on social interactions in autism, most of it concerns the deficits in social interactions. These deficits also dominate the diagnostic criteria of the autism spectrum (APA, 2013). Consequently, many studies have largely documented the issue of autistic children’s deficits in social interactions, but do not provide information on their *need* for social interaction (for a review, Gates et al., 2017; Wolstencroft et al., 2018). Like the one by Pellicano et al. (2020), our study shows that social interactions are indeed important for autistic children and could offer a new way to approach this issue, beyond the deficits. In this regard, interestingly, autistic children identified board games as a stress regulator. They could be relevant to consider, in order to maintain and foster social interactions in autistic children’s lives, because they seem to play a role in these children’s well-being and quality of life.

### Are Autistic Interests Facilitators or Obstacles?

Several studies support the view that intense interests may be associated with well-being in autism (Courchesne et al., 2020; Davey, 2020; Grove et al., 2018; Jacques et al., 2018; South & Sunderland, 2020; Warren et al., 2020). During the pandemic, spending time on their interests was characteristic of many children and adolescents. Tardif-Grenier et al. (2021) showed that spending more time on a cellphone was associated with reduced anxiety and that starting a new hobby was related to lower levels of depressive symptoms. In the present study, interests were associated with reduced stress by autistic children. They identified three activities, namely reading -an interest also highlighted as important by parents of autistic children in Nowell et al. (2020) study, board games, and electronics. In fact, almost all the children in our study emphasized the importance of electronic tools for their well-being. Although half of the parents identified too much access to electronics as a major barrier, two thirds had implemented broader access to technologies to accommodate their children. Access to electronics was also the third of 15 items identified by the parents as helping their children to cope. These results show that parents recognize the importance of interests in their autistic children’s lives. However, parents may benefit from increased knowledge of how to incorporate their children’s interests in their daily lives. Also, considering their early interest in technology (Larose et al., 2021), the way to positively integrate technology into the daily life of autistic children should be better understood (Laurie et al., 2019).

### Recommendations for Better Organizing Services for Autistic Children in an Emergency

Parents made specific recommendations on how to provide services to autistic children during the pandemic. These recommendations can guide the services offered in any emergency situation, with guidelines to facilitate their implementation. First, as pointed out by Aishworiya and Kang (2020), the various service providers (education and healthcare) and the government need to work together to find appropriate ways to meet the needs of autistic children and their families. To this effect, autism experts should be included in the decisions concerning service organization in the event of an emergency such as the current pandemic. Moreover, government authorities should develop guidelines to support assessment and intervention services for autistic children and to make the information available (Aishworiya & Kang, 2020). For example, several local initiatives, such as guidelines and newsletters for families, were developed but would benefit from being more systematic. Second, specialized service providers need to be better trained in virtual assessment and intervention to meet autistic children’s needs in times of emergency. In this regard, access to services can be improved through the use of telehealth platforms (Ameis et al., 2020; Jeste et al., 2020; Narzisi, 2020; Pellicano & Stears, 2020; Wagner et al., 2020), again supported by guidelines [e.g., virtual assessment of autism, INESSS (2020)]. In addition to providing services to parents, telehealth services can also benefit some autistic persons directly (Newbutt et al., 2020). Third, specialized services should take into account the needs and well-being not only of the children, but also of the entire families (Aishworiya & Kang, 2020; Dhiman et al., 2020).

## Limitations

Although our sample included children of all ages and allowed the collection of qualitative and quantitative information, the number of participants remained small, particularly at preschool age. It was thus difficult to perform analyses by subgroups to assess specificities in the needs identified across developmental stages. That being said, as suggested by Colizzi et al. (2020), who reported that 93.9% of parents found this time very difficult, the situation may have been less challenging for parents of older children.

We did not collect information about the specifiers associated with the autism diagnosis or the presence of comorbidities, and there was no formal verification of the diagnosis. Also, as in other studies (Adams et al., 2021; Alhuzimi, 2021; Colizzi et al., 2020; Dhiman et al., 2020), we developed an in-house questionnaire. Although it was developed and revised by clinical and research experts, it has not been validated yet. Nonetheless, it still provided a portrait of the experience of autistic children and their families because we surveyed the parents directly, a methodology used in several previous studies on autism (e.g. Hodgetts et al., 2015; Lai & Weiss, 2017; McConachie et al., 2018; Pejovic-Milovancevic et al., 2018; Płatos & Pisula, 2019; Trigueros, 2018; Twoy et al., 2007), as well as the autistic children themselves. The questionnaire also made it possible to obtain recommendations for services and governments. In this regard, some studies have shown that the perspective of parents can be an important source of information for identifying priorities for assessment and intervention services in a crisis situation (Twoy et al., 2007; White et al., 2021). Even with some limitations (e.g., some children needed help to complete the questionnaire), our study went further by directly surveying children to gather their perspectives.

Despite our efforts to reach a diversity of families via associations and by offering different modalities to complete the questionnaire, the sample was not representative of the diversity of demographic and socio-ethnic profiles and family structures. Consequently, it was not representative of the reality of the most vulnerable families (e.g., low income and education, recent migratory experience), or of parents and children who experienced the most stress during this period. For example, in Suffren et al. (2021), families with lower income and education levels had more concerns about the pandemic. This may be an explanatory factor for the absence of relationships between clinical and socio-demographic characteristics and the presence of concerns about the child's behaviors and difficulties in behavior management. However, one of the main contributions of the study concerned the parents’ recommendations, which can be informative for a large spectrum of families, by adapting them to all the realities, including those who have fewer resources and who generally face more barriers to accessing healthcare (Aishworiya & Kang, 2020; Pellicano & Stears, 2020). Finally, in the future, researchers could probe the reality of autistic children and their families in other countries during the first wave of the pandemic, in order to collect other recommendations to better support them.

## Conclusion

Our study adds to the collective effort to improve services for autistic children and their families during crises and emergency situations. One of its original contributions was to consider perspectives and recommendations from the autistic children themselves, in addition to those of their parents. Contrary to popular belief, autistic children and their parents identified social isolation and lack of socialization, respectively, as one of the main difficulties faced during the pandemic. Our study also highlighted the need to consider the child’s autistic characteristics and interests to implement emergency accommodations and services, while avoiding service disruptions.

## Supplementary Information

Below is the link to the electronic supplementary material.

Supplementary file1 (PDF 340 kb)

## Data Availability

The datasets during and/or analyzed during the current study available from the corresponding author on reasonable request. The datasets supporting the conclusions of this article are included within the article and its supplementary files.

## Footnotes

1 The participants’ quotations are translations from French.

## References

[CR1] Adams EL, Smith D, Caccavale LJ, Bean MK (2021). Parents are stressed! Patterns of parent stress across COVID-19. Frontiers in Psychiatry.

[CR2] Aishworiya R, Kang YQ (2020). Including children with developmental disabilities in the equation during this COVID-19 pandemic. Journal of Autism and Developmental Disorders.

[CR3] Alhuzimi T (2021). Stress and emotional wellbeing of parents due to change in routine for children with Autism Spectrum Disorder (ASD) at home during COVID-19 pandemic in Saudi Arabia. Research in Developmental Disabilities.

[CR4] Ameis SH, Lai M-C, Mulsant BH, Szatmari P (2020). Coping, fostering resilience, and driving care innovation for autistic people and their families during the COVID-19 pandemic and beyond. Molecular Autism.

[CR5] APA (2013). Diagnostic and statistical manual of mental disorders (DSM-5).

[CR6] Bardin L (1993). L'analyse de contenu.

[CR7] Berard M, Rattaz C, Peries M, Loubersac J, Munir K, Baghdadli A (2021). Impact of containment and mitigation measures on children and youth with ASD during the COVID-19 pandemic: Report from the ELENA cohort. Journal of Psychiatric Research.

[CR8] Bueno-Notivol J, Gracia-García P, Olaya B, Lasheras I, López-Antón R, Santabárbara J (2021). Prevalence of depression during the COVID-19 outbreak: A meta-analysis of community-based studies. International Journal of Clinical and Health Psychology.

[CR9] Calvano C, Engelke L, Di Bella J, Kindermann J, Renneberg B, Winter SM (2021). Families in the COVID-19 pandemic: Parental stress, parent mental health and the occurrence of adverse childhood experiences—results of a representative survey in Germany. European Child & Adolescent Psychiatry.

[CR10] Chanchlani N, Buchanan F, Gill PJ (2021). Les effets indirects de la COVID-19 sur la santé des enfants et des jeunes. CMAJ.

[CR11] Colizzi M, Sironi E, Antonini F, Ciceri ML, Bovo C, Zoccante L (2020). Psychosocial and behavioral impact of COVID-19 in autism spectrum disorder: An online parent survey. Brain Sciences.

[CR12] Corbett BA, Muscatello RA, Klemencic ME, Schwartzman JM (2021). The impact of COVID-19 on stress, anxiety, and coping in youth with and without autism and their parents. Autism Research.

[CR13] Courchesne V, Langlois V, Gregoire P, St-Denis A, Bouvet L, Ostrolenk A, Mottron L (2020). Interests and strengths in autism, useful but misunderstood: A pragmatic case-study. Frontiers in Psychology.

[CR14] Davey L (2020). Using the special interests of autistic children to facilitate meaningful engagement and learning. Good Autism Practice (GAP).

[CR15] Dhiman S, Sahu PK, Reed WR, Ganesh GS, Goyal RK, Jain S (2020). Impact of COVID-19 outbreak on mental health and perceived strain among caregivers tending children with special needs. Research in Developmental Disabilities.

[CR16] Eshraghi AA, Li C, Alessandri M, Messinger DS, Eshraghi RS, Mittal R, Armstrong FD (2020). COVID-19: Overcoming the challenges faced by individuals with autism and their families. The Lancet Psychiatry.

[CR17] Fegert JM, Vitiello B, Plener PL, Clemens V (2020). Challenges and burden of the Coronavirus 2019 (COVID-19) pandemic for child and adolescent mental health: A narrative review to highlight clinical and research needs in the acute phase and the long return to normality. Child and Adolescent Psychiatry and Mental Health.

[CR18] Garcia JM, Lawrence S, Brazendale K, Leahy N, Fukuda D (2020). Brief report: The impact of the COVID-19 pandemic on health behaviors in adolescents with Autism Spectrum Disorder. Disability and Health Journal.

[CR19] Gates JA, Kang E, Lerner MD (2017). Efficacy of group social skills interventions for youth with autism spectrum disorder: A systematic review and meta-analysis. Clinical Psychology Review.

[CR20] Grove R, Hoekstra RA, Wierda M, Begeer S (2018). Special interests and subjective wellbeing in autistic adults. Autism Research.

[CR21] Hodgetts S, Zwaigenbaum L, Nicholas D (2015). Profile and predictors of service needs for families of children with autism spectrum disorders. Autism.

[CR22] Institut national d'excellence en santé et en services sociaux (INESSS).(2020). COVID-19 et Services de réadaptation essentiels pour les enfants âgés de 0 à 18 ans ayant une DP, une DI ou un TSA. Retrieved March 2020, from https://www.inesss.qc.ca/fileadmin/doc/INESSS/COVID-19/COVID-19_DPDITSA_Pediatriques.pdf.

[CR23] Jacques C, Courchesne V, Meilleur A-AS, Mineau S, Ferguson S, Cousineau D, Labbe A, Dawson M, Mottron L (2018). What interests young autistic children? An exploratory study of object exploration and repetitive behavior. PLoS ONE.

[CR24] Jeste S, Hyde C, Distefano C, Halladay A, Ray S, Porath M, Wilson R, Thurm A (2020). Changes in access to educational and healthcare services for individuals with intellectual and developmental disabilities during COVID-19 restrictions. Journal of Intellectual Disability Research.

[CR25] Kendall T, Megnin-Viggars O, Gould N, Taylor C, Burt LR, Baird G (2013). Management of autism in children and young people: Summary of NICE and SCIE guidance. BMJ.

[CR26] Kunda M (2013). Visual problem solving in autism, psychometrics, and AI: The case of the Raven's progressive matrices intelligence test.

[CR27] Kunda, M., & Goel, A. K. (2008). How thinking in pictures can explain many characteristic behaviors of autism. Development and Learning, 2008. ICDL 2008. In *7th IEEE international conference on development and learning*. 10.1109/DEVLRN.2008.4640847.

[CR28] Lai JK, Weiss JA (2017). Priority service needs and receipt across the lifespan for individuals with autism spectrum disorder. Autism Research.

[CR29] Larose V, Sotelo K, Mottron L, Jacques C (2021). Initial development of a questionnaire about parents’ perspectives on the strengths and interests of autistic preschoolers. Canadian Journal of Behavioural Science/revue Canadienne Des Sciences Du Comportement.

[CR30] Laurie MH, Warreyn P, Uriarte BV, Boonen C, Fletcher-Watson S (2019). An international survey of parental attitudes to technology use by their autistic children at home. Journal of Autism and Developmental Disorders.

[CR31] Luthar SS, Cicchetti D, Becker B (2000). The construct of resilience: A critical evaluation and guidelines for future work. Child Development.

[CR32] McConachie H, Livingstone N, Morris C, Beresford B, Le Couteur A, Gringras P, Garland D, Jones G, Macdonald G, Williams K (2018). Parents suggest which indicators of progress and outcomes should be measured in young children with autism spectrum disorder. Journal of Autism and Developmental Disorders.

[CR33] McCubbin HI, Thompson EA, Thompson AI, Futrell JA (1999). The dynamics of resilient families.

[CR34] Ministère de l'Éducation et du Développement de la petite enfance, Nouveau Brunswick (MEDPENDB). (2020). Stratégies gagnantes pour gérer les changements durant la COVID-19: Un guide pour les familles d'enfants ayant un trouble du spectre de l'autisme ou des défis de développement neurologique. Retrieved April 2020, from https://jemeduque.ca/images/PAA_RessourcesFamille_FR.pdf.

[CR35] Mukamurera J, Lacourse F, Couturier Y (2006). Des avancées en analyse qualitative: Pour une transparence et une systématisation des pratiques. Recherches Qualitatives.

[CR36] Narzisi A (2020). Phase 2 and later of COVID-19 lockdown: Is it possible to perform remote diagnosis and intervention for autism spectrum disorder? An online-mediated approach. Journal of Clinical Medicine.

[CR37] NCTSN. (2020). Parent/caregiver guide to helping families cope with the coronavirus disease 2019 (COVID-19). Retrieved March 2020, from https://echoautism.org/wp-content/uploads/2020/03/National-Child-Traumatic-Stress-Network-Guide-to-help-families-cope-with-COVID_19.pdf.

[CR38] Neece C, McIntyre LL, Fenning R (2020). Examining the impact of COVID-19 in ethnically diverse families with young children with intellectual and developmental disabilities. Journal of Intellectual Disability Research.

[CR39] Newbutt N, Schmidt MM, Riva G, Schmidt C (2020). The possibility and importance of immersive technologies during COVID-19 for autistic people. Journal of Enabling Technologies.

[CR40] Nonweiler J, Rattray F, Baulcomb J, Happé F, Absoud M (2020). Prevalence and associated factors of emotional and behavioural difficulties during COVID-19 pandemic in children with neurodevelopmental disorders. Children.

[CR41] Nowell KP, Bernardin CJ, Brown C, Kanne S (2020). Characterization of special interests in autism spectrum disorder: A brief review and pilot study using the special interests survey. Journal of Autism and Developmental Disorders.

[CR42] O’Connor C, Joffe H (2020). Intercoder reliability in qualitative research: Debates and practical guidelines. International Journal of Qualitative Methods.

[CR43] Oomen D, Nijhof AD, Wiersema JR (2021). The psychological impact of the COVID-19 pandemic on adults with autism: A survey study across three countries. Molecular Autism.

[CR44] Pauzé R, Petitpas J (2013). Evaluation du fonctionnement familial: État des connaissances. Thérapie Familiale.

[CR45] Pejovic-Milovancevic M, Stankovic M, Mitkovic-Voncina M, Rudic N, Grujicic R, Herrera AS, Stojanovic A, Nedovic B, Shih A, Mandic-Maravic V (2018). Perceptions on support, challenges and needs among parents of children with autism: The Serbian experience. Psychiatria Danubina.

[CR46] Pellicano E, Stears M (2020). The hidden inequalities of COVID-19.

[CR47] Pellicano, L., Brett, S., Den Houting, J., Heyworth, M., Magiati, I., Steward, R., Urbanowicz, A., & Stears, M. (2020). “I want to see my friends”: The everyday experiences of autistic people and their families during COVID-19. Retrieved May 2020, from https://research-management.mq.edu.au/ws/files/129511617/129481673.pdf.

[CR48] Płatos M, Pisula E (2019). Service use, unmet needs, and barriers to services among adolescents and young adults with autism spectrum disorder in Poland. BMC Health Services Research.

[CR49] Roberts K, Dowell A, Nie J-B (2019). Attempting rigour and replicability in thematic analysis of qualitative research data; A case study of codebook development. BMC Medical Research Methodology.

[CR50] Salt, M., Soliman, P., & Kata, A. (2020). Sondage canadien sur l’évaluation des besoins en autisme lors d’une pandémie [Rapport v.1.0]. Autism Speaks Canada.

[CR51] Shaffer, D. R., Wood, E., & Willoughby, T. (2002). *Developmental psychology: Childhood and adolescence* (1st Canadian ed.). Thomson Nelson, Scarborough, ON.

[CR52] Simonoff E, Pickles A, Charman T, Chandler S, Loucas T, Baird G (2008). Psychiatric disorders in children with autism spectrum disorders: Prevalence, comorbidity, and associated factors in a population-derived sample. Journal of the American Academy of Child & Adolescent Psychiatry.

[CR53] South G, Sunderland N (2020). Finding their place in the world: What can we learn from successful Autists’ accounts of their own lives?. Disability & Society.

[CR54] Spain D, Mason D, Capp SJ, Stoppelbein L, White SW, Happé F (2021). “This may be a really good opportunity to make the world a more autism friendly place”: Professionals’ perspectives on the effects of COVID-19 on autistic individuals. Research in Autism Spectrum Disorders.

[CR55] Spinelli M, Lionetti F, Pastore M, Fasolo M (2020). Parents' stress and children's psychological problems in families facing the COVID-19 outbreak in Italy. Frontiers in Psychology.

[CR56] Suffren S, Dubois-Comtois K, Lemelin J-P, St-Laurent D, Milot T (2021). Relations between child and parent fears and changes in family functioning related to COVID-19. International Journal of Environmental Research and Public Health.

[CR57] Tardif-Grenier K, Archambault I, Dupéré V, Marks AK, Olivier E (2021). Canadian adolescents’ internalized symptoms in pandemic times: Association with sociodemographic characteristics, confinement habits, and support. Psychiatric Quarterly.

[CR58] Trigueros, A. F. (2018). Using parent-identified strengths of autistic children to advance strength-based intervention [Doctoral dissertation]. Walden University. Retrieved from https://scholarworks.waldenu.edu/cgi/viewcontent.cgi?article=7082&context=dissertations.

[CR59] Twoy R, Connolly PM, Novak JM (2007). Coping strategies used by parents of children with autism. Journal of the American Academy of Nurse Practitioners.

[CR60] UH. (2020). Resources for families of children with autism spectrum disorder during COVID-19. Retrieved April 2020, from https://uh.edu/education/remote-learning/sparc-covid-19-resource.pdf.

[CR61] Wagner L, Corona LL, Weitlauf AS, Marsh KL, Berman AF, Broderick NA, Francis S, Hine J, Nicholson A, Stone C (2020). Use of the TELE-ASD-PEDS for autism evaluations in response to COVID-19: Preliminary outcomes and clinician acceptability. Journal of Autism and Developmental Disorders.

[CR62] Wallace, S., Guldberg, K., & Bailey, A. (2019). A research review on autism. Eteva, Mäntsälä, Finland. Retrieved from https://www.eteva.fi/globalassets/tiedostot/tiedostot-eteva/oppaat-ja-julkaisut/a-research-review-on-autism.pdf.

[CR63] Warren N, Eatchel B, Kirby AV, Diener M, Wright C, D’Astous V (2020). Parent-identified strengths of autistic youth. Autism.

[CR64] Waters, V., Sam, A., Steinbrenner, J., Perkins, Y., Dees, B., Tomaszewski, B., Rentschler, L., Szendrey, S., McIntyre, N., & White, M. (2020). Supporting individuals with autism through uncertain times. http://greenedd.org/atrium/wp-content/uploads/Supporting-Individuals-with-Autism-through-Uncertian-Times-Full-Packet.pdf.

[CR65] White SW, Ollendick T, Albano AM, Oswald D, Johnson C, Southam-Gerow MA, Kim I, Scahill L (2013). Randomized controlled trial: Multimodal anxiety and social skill intervention for adolescents with autism spectrum disorder. Journal of Autism and Developmental Disorders.

[CR66] White LC, Law JK, Daniels AM, Toroney J, Vernoia B, Xiao S, Feliciano P, Chung WK (2021). Brief report: Impact of COVID-19 on individuals with ASD and their caregivers: A perspective from the SPARK cohort. Journal of Autism and Developmental Disorders.

[CR67] Wolstencroft J, Robinson L, Srinivasan R, Kerry E, Mandy W, Skuse D (2018). A systematic review of group social skills interventions, and meta-analysis of outcomes, for children with high functioning ASD. Journal of Autism and Developmental Disorders.

