# Supplementary material for: Experience of Autistic Children and Their Families During the Pandemic: From Distress to Coping Strategies
Source: J Autism Dev Disord. 2021 Aug 27;52(8):3626–38. doi: 10.1007/s10803-021-05233-z (PMC8391854; doi:10.1007/s10803-021-05233-z)
Supplement: Supplementary file 1 — Supplementary file1 (PDF 340 kb) [file 10803_2021_5233_MOESM1_ESM.pdf]

Supplementary Material

Table 1

Associations Between Being Concerned About the Child's Development, Child's behaviors Are Easy to Manage on a Daily Basis and Stress Level

	1	2	3	4	5	6	7	8
**1**	-							
**2**	**-.503****	-						
**3**	**.313****	**-.297****	-					
**4**	-.171	.145	**-.394****	-				
**5**	**.251****	**-.215***	**.544****	**-.333****	-			
**6**	**.379****	**-.392****	**.690****	**-.325****	**.773****	-		
**7**	-.051	.083	-.081	.141	-.110	-.131	-	
**8**	**.225***	-.183	**.271****	**-.301****	**.346****	**.408****	**-.332****	-

*$p < .05$ **$p < .01$.

1. I am concerned about my child's development.
2. My child's behaviors are easy to manage on a daily basis.
3. I consider the pandemic period to be a great source of stress for my child.
4. I see the pandemic period as an opportunity to enjoy quality time with entourage.
5. I consider the pandemic period to be a great source of stress for me.
6. I consider the pandemic period to be a great source of stress for our family.
7. Over time, the situation becomes less stressful (for me, my family, my child).
8. Over time, the situation becomes more stressful (for me, my family, my child).

Note. This table demonstrates the associations between concerned about the child's development, ease of managing behaviors and stress level (n=109)

Table 2
Associations Between Being Concerned About the Child's Development, Child's Behaviors Are Easy to Manage on a Daily Basis and Sociodemographic and Clinical Characteristics

	1	2	3	4	5	6	7	8	9
**1**	-								
**2**	**,534****	-							
**3**	**-,322****	**-,318****	-						
**4**	**-,338****	**-,503****	**,519****	-					
**5**	-0,113	-0,041	**,192***	0,137	-				
**6**	-0,017	0,150	0,071	-0,007	0,015	-			
**7**	-0,084	**-,216***	0,033	0,102	0,116	0,018	-		
**8**	-0,112	-0,132	0,185	**,273****	**,227***	-0,060	0,122	-	
**9**	-0,018	0,057	-0,092	-0,042	**,253****	-0,032	-0,073	**,192***	-

*p < .05 **p < .01.

1. I was concerned about my child's development.
2. I am concerned about my child's development.
3. My child's behaviors were easy to manage on a daily basis.
4. My child's behaviors are easy to manage on a daily basis.
5. Age of child.
6. Number of siblings.
7. Family income.
8. Level of education (parent 1).
9. Level of education (parent 2).

Note. This table demonstrates the associations between concerned about the child's development, ease of managing behaviors and sociodemographic and clinical characteristics (n=109).

Table 3

Associations Between Being Concerned About the Child's Development, Child's Behaviors Are Easy to Manage on a Daily Basis and Facilitating Factors (part 1)

	1	2	3	4	5	6	7	8	9	10	11	12	13	14	15	16	17	18	19	20
**1**	-																			
**2**	**.534****	-																		
**3**	**-.322****	**-.318****	-																	
**4**	**-.338****	**-.503****	**.519****	-																
**5**	.024	.179	-.057	-.081	-															
**6**	.046	.110	-.105	-.095	**.288****	-														
**7**	-.061	-.078	.019	-.018	**.317****	**.247****	-													
**8**	**.389****	**.266****	**-.194***	-.176	.084	.081	.108	-												
**9**	**.194***	**.285****	-.152	**-.318****	.129	**.254****	.135	**.316****	-											
**10**	.172	**.211***	**-.463****	**-.523****	-.018	.034	.002	**.216***	.244*	-										
**11**	.111	.024	-.121	-.043	.072	**.273****	.083	.016	.024	.091	-									
**12**	**.239***	**.250****	**-.367****	**-.386****	.094	**.219***	.134	.173	**.358****	**.270****	**.291****	-								
**13**	.055	.104	-.143	-.002	.006	.175	.069	.048	.069	.136	.161	.113	-							
**14**	-.047	-.115	.097	.056	.046	-.115	.124	.063	.048	-.089	-.168	.110	-.047	-						
**15**	-.032	-.022	-.134	-.005	-.023	.003	**.308****	.072	.025	.109	.127	.037	-.067	.101	-					
**16**	**-.301****	**-.220***	.163	.133	.034	.016	.043	.146	.001	-.042	-.133	**-.281****	**-.192***	**.287****	.063	-				
**17**	-.074	-.086	.102	.109	.101	.083	.016	-.004	.071	-.058	.132	-.017	-.181	.183	.056	**.226***	-			
**18**	.046	-.098	**-.213***	-.017	.141	.018	.104	.081	.095	.160	-.090	-.091	-.094	.174	.160	**.254****	-.037	-		
**19**	-.001	-.072	-.105	.009	**.239***	.100	**.247****	.169	.016	.076	.031	-.052	-.161	.174	**.200***	**.214***	**.203***	.182	-	
**20**	-.078	-.035	-.076	.036	-.082	**.235***	-.087	-.153	-.021	-.111	-.009	.048	-.053	-.120	.090	.094	-.015	-.002	-.002	-

$^{*}p < .05$ $^{**}p < .01$.

1. I was concerned about my child's development.
2. I am concerned about my child's development.
3. My child's behaviors were easy to manage on a daily basis.
4. My child's behaviors are easy to manage on a daily basis.
5. I have time for myself.
6. I have access to information on COVID-19.
7. I integrated my personal interests into my routine.
8. I understand my child's needs.
9. I feel competent and able to help my child.
10. My child has an easy temperament.
11. My child is heading towards his interests.
12. My child is able to take care of himself.
13. My child is looking on information about COVID-19
14. Establishing a routine with my child.
15. Using my child's strengths.
16. The use of visual representations (image, pictogram, drawing, list) with my child.
17. Organizing my child's environment
18. Setting up learning activities with my child.
19. Setting up physical activities for my child.
20. My child attended emergency childcare service.

Note. This table demonstrates the associations between concerned about the child's development, ease of managing behaviors and facilitating factors (n=109).

Table 3

Associations Between Being Concerned About the Child's Development, Child's Behaviors Are Easy to Manage on a Daily Basis and Facilitating Factors (part 2)

	**1**	**2**	**3**	**4**	**5**	**6**	**7**	**8**	**9**	**10**	**11**	**12**	**13**	**14**	**15**	**16**	**17**	**18**
**1**	-																	
**2**	**.534****	-																
**3**	**-.322****	**-.318****	-															
**4**	**-.338****	**-.503****	**.519****	-														
**5**	-.006	-.020	.091	.064	-													
**6**	.079	.075	-.116	-.142	-.081	-												
**7**	**.268****	**.264****	-.072	**-.204***	,146	**.259****	-											
**8**	.066	.027	-.071	-.118	-.054	.055	.147	-										
**9**	.153	.084	**-.240***	**-.237***	-.034	**.241***	.151	**.216***	-									
**10**	.159	.174	-.035	-.092	.103	.147	**.343****	**.209***	.140	-								
**11**	**.206***	**.209***	-.113	**-.210***	-.070	**.286****	**.482****	**.268****	**.296****	**.626****	-							
**12**	.101	.043	-.169	-.168	-.050	.114	.100	.185	.034	.101	.046	-						
**13**	.119	.035	.004	-.058	-.124	.151	.099	**.212***	.092	.045	.091	.030	-					
**14**	.052	.062	-.011	-.079	-.085	.044	**.300****	.111	-.017	.020	.165	.085	**.269****	-				
**15**	.093	.001	-.164	-.092	-.090	.035	.022	.036	-.036	.042	.124	.101	**.235***	**.279****	-			
**16**	.098	-.037	-.093	.005	-.122	.013	**.193***	.134	-.023	.138	.184	-.053	.086	**.361****	.100	-		
**17**	.127	.054	-.120	-.111	-.062	**.196***	.058	**.679****	**.288****	**.203***	**.247****	**.259****	**.251****	.077	.082	.096	-	
**18**	**.228***	**.257****	-.096	-.116	-.087	**.254****	**.247****	-.073	.154	**.520****	**.423****	-.106	.016	.016	.077	.185	.025	-

*$p < .05$ **$p < .01$.

1. I was concerned about my child's development.
2. I am concerned about my child's development.
3. My child's behaviors were easy to manage on a daily basis.
4. My child's behaviors are easy to manage on a daily basis.
5. My child having a job.
6. Access to electronic devices for my child.
7. Free time for my child.
8. Virtual contact with significant relatives.
9. Access to information about COVID-19.
10. Decrease in daily responsibilities towards academic demands.
11. The reduction of pressure related to academic performance.
12. Virtual contact with teachers.
13. Good communication between parents.
14. Setting up family activities.
15. Good relationships between children in the family
16. The possibility of spending time together
17. Virtual contact with family members.
18. The decrease in responsibilities in general.

Note. This table demonstrates the associations between being concerned about the child's development, ease of managing behaviors and facilitating factors (n=109).

Table 4

Associations Between Being Concerned About the Child's Development, Child's Behaviors are Easy to Manage on a Daily Basis and Barriers

	1	2	3	4	5	6	7	8	9	10	11	12	13	14	15	16	17	18	19	20
1	-																			
2	.534**	-																		
3	-.322**	-.318**	-																	
4	-.338**	-.503**	.519**	-																
5	-.084	-.098	.042	.276**	-															
6	-.193*	-.041	.007	.130	.188	-														
7	-.172	-.164	-.098	.203*	-.030	.066	-													
8	-.152	-.130	.051	.006	.044	-.178	-.213*	-												
9	-.063	-.052	-.172	-.079	.135	-.125	-.034	.137	-											
10	-.163	-.187	.037	.005	.239*	.021	-.071	.160	.329**	-										
11	.135	-.134	-.057	.075	.008	-.021	.115	.083	.046	.199*	-									
12	-.007	.133	-.044	.012	.146	.165	.317**	-.188	.001	-.130	-.082	-								
13	-.417**	-.462**	.264**	.333**	.164	.060	.072	.058	.153	.056	.032	-.047	-							
14	-.187	-.326**	.263**	.276**	.142	.047	.046	.158	.090	.062	.134	-.145	.435**	-						
15	-.069	-.144	-.199*	.035	.340**	.090	.131	-.040	.287**	.175	-.087	.222*	.180	.060	-					
16	-.178	-.292**	.180	.261**	.029	.144	.139	.111	-.079	-.053	.096	.073	.361**	.514**	.117	-				
17	-.436**	-.434**	.222*	.241*	.168	.016	.159	.061	.078	.080	.129	-.006	.324**	.139	.182	.203*	-			
18	-.162	-.108	.096	.092	.182	.075	-.066	.178	.162	.090	.254**	.015	.236*	.355**	.226*	.203*	.282**	-		
19	.042	.056	-.154	-.080	.029	.104	.038	.065	.010	.209*	-.130	.008	-.100	-.118	.158	-.012	-.024	.117	-	
20	-.080	-.033	-.002	.056	.186	.032	-.102	.154	.170	.175	.044	.004	.209*	.112	.274**	.062	.049	.191*	.254**	-

*$p < .05$ **$p < .01$.

1. I was concerned about my child's development.
2. Have concerned about my child's development.
3. My child's behaviors were easy to manage on a daily basis.
4. My child's behaviors are easy to manage on a daily basis.
5. My child has no more routine.
6. My child has late and / or disturbed sleep.
7. My child attends emergency childcare service.
8. My child is moving more towards his particular interests.
9. My child has too much free time.
10. My child has access to electronic devices.
11. My child has access to information about COVID-19.
12. I work in essential services several hours a week.
13. I feel helpless facing my child's needs
14. I need support to overcome some personal vulnerabilities.
15. I cannot give my child enough time.
16. I need to be better informed and better equipped.
17. We avoid going outside.
18. We are more isolated from our loved ones.
19. It is necessary to pursue academic goals at a distance.
20. There is a decrease in interventions by school personne

Note. This table demonstrates the associations between concerned about the child's development, ease of managing behaviors and barriers (n=109).

Table 5

Top 3 Facilitating Factors by Age Groups

	n	%
**Prescolar (6 years and less) (N=10)**		
**Personal facilitators**		
I understand my child's needs	7	70
I feel competent and able to help my child	5	50
I have access to information on COVID-19	3	30
**Environmental facilitators**		
The use of visual representations (images, pictograms, etc.)	7	70
Establishing a routine with my child	6	60
Using my child's strength	5	50
Setting up physical activities for my child	5	50
**Family facilitators**		
Good communication between parents	10	100
The possibility of having time spent together	7	70
Good understanding between the children of the family	6	60
**Scolar (6 years to 12 years) (N=63)**		
**Personal facilitators**		
I understand my child's needs	47	74,60
My child is able to take care of himself	30	47,62
I feel competent and able to help my child	25	39,68
**Environmental facilitators**		
Establishing a routine with my child	46	73,02
Decrease in daily responsibilities towards academic demands	34	53,97
Setting up learning activities with my child	28	44,44
Using my children's strength	28	44,44
**Family facilitators**		
The possibility of having time spent together	41	65,08
Good communication between parents	38	60,32
The establishment of family activities	32	50,79

**High school (13 years and more) (N=37)**		
**Personal facilitators**		
I understand my child's needs	26	70,27
My child is able to take care of himself	25	67,57
My child is heading towards his interests	17	45,95
**Environmental facilitators**		
Establishing a routine with my child	22	59,46
Access to electronic devices for my child	18	48,65
Decrease in daily responsibilities towards academic demands	17	45,95
**Family facilitators**		
Good communication between parents	21	56,76
The possibility of having time spent together	20	54,05
The reduction of responsibilities in general	18	48,65

Note. This table demonstrates the top 3 facilitating factors during the pandemic by age group, as endorsed by the respondents (n=109) in multiple-choice questions. The respondents could select all the answers that they thought had a positive impact on their capacity to cope with the pandemic.

Table 6
Facilitating Factors

	n	%
**Personal facilitators**		
I have time for myself	21	19.27
I have access to information on COVID-19	37	33.94
I integrated my personal interests into my routine	23	21.10
I understand my child's needs	80	73.39
I feel competent and able to help my child	43	33.45
My child has an easy temperament	33	30.28
My child is heading towards his interests	39	35.78
My child is able to take care of himself	57	52.29
My child is looking for information about COVID-19	10	9.17
**Environmental facilitators**		
Establishing a routine with my child	73	66.97
Use of my child's strengths	44	40.37
The use of visual representations (image, pictogram, drawing, list) with my child	43	39.45
Organizing my child's environment	41	37.61
Setting up learning activities with my child	37	33.94
Setting up physical activities for my child	37	33.94
My child attended emergency child care	3	2.75
My child having a job	1	0.92
Access to electronic devices for my child	45	41.28
Free time for my child	33	30.28
Virtual contact with significant relatives	26	23.85
Access to information about COVID-19	12	11.01
Decrease in daily responsibilities towards academic requirements	51	46.79
The reduction of pressure related to academic performance	38	34.86
Virtual contact with teachers	23	21.10
**Family facilitators**		
Good communication between parents	68	62.39
Setting up family activities	48	44.04
Good relationships between children in the family	51	46.79
The possibility of spending time together	67	61.47
Virtual contact with family members	32	29.36
The reduction of responsibilities in general	49	44.95

Note. This table demonstrates the facilitating factors during the pandemic, as endorsed by the respondents (n=109) in multiple-choice questions. The respondents could select all the answers that they thought had a positive impact on their capacity to cope with the pandemic.

Table 7

Top 3 Barriers by Age Groups

**Prescolar (6 years and less)**	***n***	**%**
We are more isolated from our loved ones	6	60
My child has late and / or disturbed sleep	5	50
We avoid going outside	5	50
**Scolar (6 years to 12 years)**		
My child has access to electronic devices	42	66,7
We are more isolated from our loved ones	40	63,49
It is necessary to pursue academic goals at a distance	36	57,14
**Highschool (13 years and more)**		
It is necessary to pursue academic goals at a distance	19	51,35
My child has access to electronic devices	18	48,65
There is a decrease in interventions by school staff	17	45,95
My child is moving more towards his particular interests	17	45,95

Note. This table demonstrates the top 3 barriers during the pandemic by age group, as endorsed by the respondents (n=109) in multiple-choice questions. The respondents could select all the answers that they thought had a negative impact on their capacity to cope with the pandemic.

Table 8
Barriers

**Barriers**	***n***	**%**
My child has no more routine	51	46.79
My child has late and / or disturbed sleep	49	44.95
My child attends emergency childcare service	5	4.59
My child works outside	0	0
My child is moving more towards his particular interests.	53	48.62
My child has too much free time	52	47.71
My child has access to electronic devices	61	55.96
My child has access to information about COVID-19	21	19.27
I work in essential services several hours a week	23	21.11
I feel helpless facing my child's needs	28	25.69
I need support to overcome some personal vulnerabilities	33	30.28
I cannot give my child enough time	35	32.11
I need to be better informed and better equipped	18	16.51
We avoid going outside	48	44.04
We are more isolated from our loved ones	60	55.05
It is necessary to pursue academic goals at a distance	56	51.38
There is a decrease in interventions by school personnel	47	43.12

Note. This table demonstrates the barriers during the pandemic, as endorsed by the respondents (n=109) in multiple-choice questions. The respondents could select all the answers that they thought had a negative impact on their capacity to cope with the pandemic. *Essential services mean services considered a priority, which remained open during the pandemic and which require an in-person presence.
